# When the present visits the past: Updating traumatic memories in social phobia

**DOI:** 10.1016/j.jbtep.2007.07.003

**Published:** 2007-12

**Authors:** Jennifer Wild, Ann Hackmann, David M. Clark

**Affiliations:** a Department of Psychology, Institute of Psychiatry, De Crespigny Park, London SE5 8AF, UK; b Department of Psychiatry, University of Oxford, Oxford OX1 2JD, UK

**Keywords:** Imagery rescripting, Imagery, Traumatic memory, Social phobia, Anxiety

## Abstract

Research suggests that distorted images of the self are common in social phobia and play a role in maintaining the disorder. The images are often linked in thematic and sensory detail to distressing memories that are clustered around the onset or worsening of the disorder. This has led to speculation about the likely benefit of working directly with these memories to improve symptoms of social phobia. In this exploratory study, we describe a process of cognitive restructuring followed by imagery rescripting to update the meanings of distressing memories and images in social phobia. We first present illustrative clinical examples and then data of 14 patients with social phobia, on whom we developed this approach. Patients attended an imagery rescripting session in which a semi-structured interview was used to identify their recurrent images, the associated memories and their meanings. Next the identified memory was evoked and elaborated. We updated the meaning of the memory by first using cognitive restructuring to arrive at new perspectives and then linking these perspectives with the memory using imagery techniques. The procedure resulted in significant within session change in beliefs, and in image and memory distress and vividness. One week later significant change was seen in social phobia cognitions and a self-report measure of social anxiety. Rescripting distressing memories in social phobia appears to be an effective way of modifying maladaptive beliefs linked to recurrent negative imagery. This paper presents our exploratory investigation of how to work with the memories and encourages more rigorous investigation in this area.

## 1 Introduction

At the heart of cognitive models of anxiety lies the idea that distorted appraisals lead to perceived threat and anxiety. These appraisals are typically described as verbal thoughts. To access highly charged appraisals, Beck (1976) stressed the importance of considering images and memories as well as verbal thoughts. In their manual on cognitive therapy for anxiety disorders, Beck, Emery, and Greenberg (1985) also note that in addition to affect and increased estimates of danger, imagery spontaneously emerges when approaching or entering a feared situation, further highlighting its relevance to anxiety. These observations are consistent with recent research on social phobia.

The text revision of the fourth edition of the Diagnostic and Statistical Manual of Mental Disorders (DSM-IV-TR; American Psychiatric Association, 2000) describes social phobia as a persistent fear of certain social or performance situations, in which the person fears that they will act in a way (or show anxiety symptoms) that will be embarrassing or humiliating. A key suggestion in the Clark and Wells (1995) cognitive model of social phobia is that when feeling threatened in social situations, patients switch to processing the self as a social object, their attention turning to internal rather than external stimuli. Consistent with this view, a number of studies have found when patients with social phobia enter social situations, or recall such situations, they experience visual and/or somatic images of themselves as an object of the scrutiny of others. They report seeing themselves as if from an observer's perspective and appearing the way they imagine they come across to other people (Coles, Turk, & Heimberg, 2002; Coles, Turk, Heimberg, & Fresco, 2001; Hackmann, Surawy, and Clark, 1998; Wells, Clark, & Ahmad, 1998). They report appraising this mental representation as mirroring a true reflection of the self, yet the image or impression is typically distorted in a negative way. Many studies (Alden & Wallace, 1995; Mulkens, de Jong, Dobbelaar, & Bogels, 1999; Rapee & Abbott, 2006; Rapee & Lim, 1992; Stopa & Clark, 1993) have shown that patients hold excessively negative self-perceptions: relative to observers, they underestimate their social performance, overestimate how visible their anxiety symptoms appear and underestimate their general attractiveness.

Rapee and Heimberg (1997) also suggest that on entering a social situation a socially phobic person forms a mental representation of their appearance and behaviour (as presumably seen by an audience) and simultaneously focuses attention onto both this internal representation and on any perceived threat in the environment. The mental representation is considered to be a loose amalgam based on a variety of inputs, including material from long term memory.

The Clark and Wells (1995) and Rapee and Heimberg (1997) models of social phobia incorporate patients’ attention to negative self-images. It is difficult to establish the exact sequence of appraisals, imagery and attentional deployment in anxiety-provoking situations as events unfold so quickly in response to perceived danger. However, research suggests that negative self-images may have a causal role in social anxiety. Hirsch, Clark, Mathews, and Williams (2003) asked patients with social phobia to hold a negative or neutral image in mind whilst having a conversation with a stranger. The conversation was videotaped and an independent assessor watched the video to rate patients’ performance. Patients felt more anxious and believed they came across more poorly when they held the negative image in mind compared to when they held the neutral image in mind. Further, the independent assessor's ratings of patients’ performance were lower in the negative image condition. These findings have been replicated with non-clinical participants who are high in social anxiety (e.g., Hirsch, Meynen, & Clark, 2004; Vassilopoulos, 2005) and in participants who are low in public speaking anxiety (Hirsch, Mathews, Clark, Williams, & Morrison, 2006). Overall, the results suggest that deliberately holding a negative image in mind (rather than a positive or more neutral image) makes participants feel more anxious, engage in more safety behaviours, believe they come across more poorly and receive poorer performance ratings by an observer.

Future studies could more closely examine whether it is only negative self-images that have these effects, or whether any anxiety provoking control image might be equally detrimental. However, the results so far suggest that negative self-images are prevalent, and play a role in maintaining social phobia. They trigger anxiety and safety behaviours, both of which can play their part in perpetuating underlying negative appraisals of social situations.

There is also some evidence that the negative self-images reported in social phobia may have input from autobiographical memory. Hackmann, Clark, and McManus (2000) carried out a study of images and their possible links to memories in this disorder. All participants reported negative, spontaneous self-imagery that was recurrent. That is, the images were relatively stable across time and in feared social situations. In addition, most recurrent images were linked to memories of adverse social events, clustered in time around the onset or worsening of the disorder. These events typically involved bullying, criticism or humiliation. The links between patients’ current imagery and earlier events were established by evoking the recurrent image, exploring its content and meaning, then enquiring when in their life the patient could remember having had a similar experience. The study suggested that the intrusive images that are characteristic of social phobia may have substantial input from events in memory, since the reported images and memories were often strikingly similar in content and meaning. Further, patients frequently described the memories as representing points at which their social phobia started or worsened. Conway, Meares, and Standart (2004) suggest that memories of negative, self defining moments can be aversive, leading to avoidance, with little opportunity for these memories to find a place in autobiographical memory. This makes them more likely to be triggered automatically with the potential to disrupt functioning. They may then be described as intrusions that are “out of context, out of proportion, or simply out of time” (Rachman, 1980).

Since such imagery appears to contain input from memory of upsetting experiences, we have previously speculated on the potential value of working directly with such memories in cognitive therapy for anxiety disorders (Hackmann, 2005). Intrusive imagery with input from memory of a traumatic event is an important focus in cognitive therapy for posttraumatic stress disorder (PTSD), for example. In the Ehlers and Clark (2000) approach to PTSD, attention is paid to deliberately access the meanings of the worst moments of the trauma through the process of reliving, and then updating these meanings with information that provides more realistic (e.g., “I did not die”) and less toxic (e.g., “It was not my fault”) appraisals of the trauma. This is accomplished using verbal and imagery techniques (Ehlers, Clark, Hackmann, McManus, & Fennell, 2005). During cognitive therapy for PTSD that involves these elements, the intrusive imagery decreases in frequency, distress and vividness (Ehlers, Hackmann, & Michael, 2004; Hackmann, Ehlers, Speckens, & Clark, 2004) and idiosyncratic belief ratings change. These results, and other observations across disorders, lead to the idea that where there is recurrent, intrusive imagery, there may be input from past experience, and furthermore, it may be productive to work directly on disturbing memories using similar techniques to those used in the treatment of PTSD.

These suggestions are not entirely novel. Throughout the history of cognitive behavioural therapy (CBT), value has been placed on the element of imaginal exposure, giving attention to images which appear to reflect input from memory. We see this, for example, in systematic desensitisation (Weitzman, 1967), imaginal flooding (Levis, 1980) and repeated reliving or imaginal exposure (Foa & Rothbaum, 1998). In addition, procedures for addressing and transforming the content or meaning of upsetting memories have been described by many (e.g., Arntz & Weertman, 1999; Beck et al., 1985; Beck, 1995; Edwards, 1990; Giesen-Bloo et al., 2006; Hackmann, 1998, 2005; Layden, Newman, Freeman, & Morse, 1993; Young, Klosko, & Weishhaar, 2003).

However, there is a relatively limited amount of information concerning the outcome of this set of therapeutic procedures studied in isolation from other treatment components. Smucker and Neiderdee (1995) report on the therapeutic effect of imagery rescripting in PTSD arising from childhood sexual abuse. A study by Hunt et al. (2006) on participants fearful of snakes found that treatment condition (cognitive imagery modification, in-vivo exposure, or minimal exposure plus relaxation) interacted with initial severity. Specifically, highly fearful participants responded better to cognitive imagery modification than to in-vivo exposure, and they found the intervention less aversive. Both the active treatment groups improved significantly more than the group provided with a minimal exposure, relaxation control procedure. Also, delivered in a single session, imagery rescripting has produced significantly greater change than verbal discussion in bulimia nervosa (Cooper, Todd, & Turner, in press, 2007; Ohanian, 2001). Grunert, Smucker, Weis, and Rusch (2003) have reported finding imagery rescripting an effective extra component in the treatment of PTSD in cases where imaginal exposure alone has not proved effective.

This Special Issue is timely as it brings together recent attempts to gather more information about the effectiveness of this type of intervention. Wheatley, Brewin, Patel, and Hackmann (this issue) describe two cases of patients with depression and intrusive memories where they obtained good results from sessions devoted exclusively to rescripting disturbing memories, and the benefits were maintained at 6 month follow-up. Arntz, Tiesema, and Kindt (this issue) present a comparison of imaginal exposure with and without imagery rescripting in the treatment of PTSD to begin the process of examining the efficacy of various elements in the treatment of disturbing memories.

Other recent studies also point to the usefulness of imagery rescripting. Weertman and Arntz (in press) report the results of a comparison of imagery rescripting of disturbing memories with more standard schema change techniques in personality disorder. They found that both types of intervention led to significant change. For patients and therapists, the preferred order involved working with the memories, then moving to more present-focused work. In another study of social phobia, Wild, Hackmann, and Clark (in press) compared a single session of imagery rescripting to a control session in which the memories were only discussed. The imagery rescripting session led to significantly greater reductions in social anxiety than the control session.

When we carried out the study reported here, there had been no previous studies on the effects of rescripting distressing memories on cognitions and behaviours specific to social phobia. However, we had used imagery rescripting with a subset of individuals in a recent randomised controlled trial of cognitive therapy for social phobia (Clark et al., 2006) and had the impression that it had been useful. The time seemed right to examine its effects (when delivered in a single session) on social phobia cognitions and behaviours. This paper allows us the opportunity to describe our procedure in detail, and present case material to illustrate the technique and its impact. Our procedure is unusual in that it involves an element of verbally challenging old appraisals and considering new ones, before consolidating the new perspectives in imagery. In this way it is similar to the Ehlers et al. (2005) approach to PTSD, which interweaves verbal and imagery techniques. Encouraged by the speed and magnitude of the changes in this small, exploratory study we have subsequently put our version of rescripting to a more rigorous test by comparing it to a control session of simply exploring the memories (Wild et al., in press). This has led to promising results with the imagery rescripting session demonstrating significantly greater improvement in social anxiety than the control session.

## 2 Description of the intervention

### 2.1 Imagery interview

A semi-structured interview (Hackmann et al., 2000) was administered immediately prior to the memory rescripting procedure. In the interview, an example of the participant's recurrent image in social situations was identified and described in the first person, present tense. The meaning of the image was elicited in relation to beliefs about the self, others and the world. The interview then identified a distressing memory linked to the recurrent image. To do this, participants were asked when they first remembered an experience that felt similar to that in their image. If several memories were elicited, the most distressing memory with the most similar meaning to the recurrent image was selected. Next the memory was brought to mind and its meaning elicited. The meanings of both the image and memory were usually very similar and participants were asked to summarise in their own words the ‘encapsulated belief’, a statement that captured both meanings.

The imagery rescripting session followed the imagery interview. It involved two components: cognitive restructuring to verbally challenge the old beliefs; and rescripting the memory to include new perspectives, help contextualise the upsetting experience, and update the patient's understanding of it. They were given a short rationale:

In the memory, it felt like (for example) people were always going to laugh at you. In your image, you have a feeling that (for example) people are about to laugh at you. The feeling is the same as in the memory. It sounds like you’re responding to people as if they are (for example) your classmates. It is possible that the memory was something that happened to you when you were younger, that does not apply to you as an adult or with other people. It is possible that what happened in the memory is not happening now, but it is like you are being haunted by the memory. You may be processing the present as though it is the same circumstance as the past memory. We need to get rid of the ghost. To do this, we’ll have a look at what your image and memory mean, then go back and look at the memory in more detail.

Patients then had a period of cognitive restructuring lasting up to 30 min to help them challenge the ‘encapsulated belief.’ Having generated the evidence for and against the patient's beliefs the therapist summarised this on the whiteboard.

The information gleaned during the verbal restructuring was often incorporated in the rescripting, which lasted approximately 45 min. Patients revisited their memory in three stages. In the first step, the patient imagined they were the age at which the event occurred, and relived it as if it happening again. Then they relived the memory at their current age, watching what happened to their younger self. They could imagine intervening at this stage if they wished. This often involved confronting critical others, and/or conveying to the younger self the alternative perspective they had come up with in the cognitive restructuring phase. Finally, they usually relived the memory again from the perspective of their younger self with their adult self in the room with them, intervening as before. This time the younger self was asked what else they might need to happen to feel better, and then to imagine this happening. The younger self often requested extra nurturing and compassion at this point.

## 3 Two case examples^1^

*John* had been a shy boy, but did well at school, and had two good friends. In his late teenage years he and his friends experimented with drugs. When he was 20 John wanted to stop taking drugs, but his friends put a lot of pressure on him to carry on, and began to bully and criticise him. They spread gossip about him, and made his life a misery. In his recurrent image John pictured himself looking acutely self-conscious, with a red face and very big ears. He also experienced himself as alone without any back-up. The associated memory was of arriving at a house where one of these friends was having a party, to which he had invited lots of people John did not know. When he arrived at the door his friend shouted out: “If that's the weird guy with the big ears tell him he is not welcome here.” At that point John felt humiliated, and believed he was ugly, unlikable and immature, beliefs that were also activated in social situations in the present.

During the verbal discussion phase, the therapist encouraged John to come up with alternative ways of seeing the situation. This included thinking of reasons why teenagers sometimes tease and try to humiliate their friends, and what this says about the teenagers who humiliated him, rather than himself. He was encouraged to think of what else he knew now about his “friends,” and to consider examples before and after this event, in which he was not humiliated or rejected by other people. In essence, the therapist helped John to distinguish between what happened and what he thought when he was young, and what he now knew about the situation. This was to help him to see the disturbing event as a time-limited experience without implications for his present or future and to generate an adult perspective that would be incorporated with the memory in the rescripting phase.

Verbal restructuring elicited the facts that his friends had both become drug addicts, and had fairly disastrous relationships with others, whilst he still had good relationships with other friends and his family, and had a nice partner and a baby.

In the imaginal rescripting he intervened in the party scene, telling his friend to back off and leave his younger self alone. When he relived the memory again as his younger self (with the adult self intervening) he was asked if there was anything else he needed to make him feel better. He chose to introduce his second “friend” into the situation, and then bring in other friends and members of his own family and the families of his two friends. When he had done this he went on to imagine himself confronting the two bullies. He asked the people who knew them all to back the bullies or himself. Slowly all the assembled family and friends lined up to back him, rather than his old “friends.” This was because these young men had become losers and upset a lot of people, whilst John had quietly got on with his own life.

John felt calmer and happier once he could view the situation in this way. Also he felt that the new image reflected current reality, whilst the old one really was a ghost from the past.

*Sarah* was a 30 year old woman who had a recurrent image in social situations of looking blank and odd with a mouth that moved with no words coming out. This image developed when she was 23 years old after an experience at work that was also linked to the onset of her social phobia. Sarah worked for a media company then. Her new manager, Stacy, called her into her office and asked, “Tell me what you’ve been working on over the last few months.” Sarah's mind went blank and a wave of fear passed through her. She could not think of what to say and this made her feel more and more anxious. She felt as though ten minutes of silence passed before she could speak at which point, she said “I’m sorry, I need to go to the toilet.” Sarah felt embarrassed and ashamed. She thought all her colleagues knew that she had rushed out of her meeting with Stacy because she was so nervous. She thought they all believed she was weird. As a result of the event, Sarah took two weeks off work and then left her job permanently. Sarah believed the event and now her image meant that she was an anxious and weird person, that people would see this and reject her.

The cognitive restructuring phase helped Sarah to look at the event in alternative ways. Her work at the media company had been during a stressful time in her life. Through guided discovery, Sarah concluded that what she perceived to be signs of being an anxious person could also be signs of transient stress. Sarah was working long hours, her father had just been hospitalised for early onset dementia, her boyfriend had split up with her and she had had to move house. Looking at the evidence for and against the belief that she is weird revealed that what she defined as weird behaviour had nothing to do with her behaviour with Stacy. Sarah thought that weird behaviour was talking nonsense like saying “pigs wear ribbons,” laughing inappropriately, wearing strange clothing, walking funny and behaving as if you are really drunk all the time. She realised she had done none of these things with Stacy. In further questioning about her work, she also remembered that the media company telephoned her 2 months after she had left and invited her to join a new project they were working on. Sarah was asked how this request fit with the belief that they perceived she was weird and anxious and wanted to reject her. She said she had never looked at the situation in this way before.

In the rescripting phase, Sarah relived the memory from the age at which it occurred. She then relived it again as a 30 year old in the office watching what happened to her 23 year old self. She said the 23 year old in her memory looked stressed and in need of reassurance rather than criticism. She noticed that in fact it did not look at all like 10 minutes had passed before she could speak, but rather seconds. She intervened at this stage and reassured the 23 year old. She spoke to her about all her worries about looking anxious, and summarised what she had learned in the cognitive restructuring phase about signs of stress. She also pointed out that her new manager did not reject her. In fact, the media company asked her to stay on for another job. She pointed out that she has lots of friends now and people do not reject her. She also looked around at other people in her office and she said she could see them getting on with their work, not at all aware of Sarah leaving the meeting with Stacy. Sarah conveyed that she had not been rejected then and even now 7 years on, is still not rejected. In the final phase of the rescripting, Sarah relived the event from the 23 year old perspective with her 30 year old self in the room. This time 23 year old Sarah had a chat with her new manager. She explained that she felt overworked and that she was also having a hard time at home. She asked for feedback. In the feedback, her new manager said that she had received very good reports about Sarah's work and that they would like to keep her on for another project.

After rescripting, Sarah said that she realised she had been the one who had created the negative image of herself. She said she could see other people did not reject her then or now and that her behaviour had not been weird but rather, understandable given the circumstances.

## 4 Development series

Next we describe the results obtained in a series of patients who received memory rescripting while we were developing the procedure.

### 4.1 Method

#### 4.1.1 Participants

Our sample consisted of 14 patients (five female) who were receiving or about to receive cognitive therapy for social phobia. Twelve were attending the Centre for Anxiety Disorders and Trauma in London and had received four sessions of treatment. Two had attended the University Psychiatry Department at the Warneford Hospital in Oxford for assessment and had not yet started treatment. They all met the criteria for social phobia on the Structured Clinical Interview for DSM IV (SCID; First, Gibbon, Williams, & Spitzer, 1995) and the social phobia module of the Anxiety Disorders Interview Schedule (ADIS; Brown, Di Nardo, & Barlow, 1994). Patients had a mean score of 84.42 (SD=17.99) on the Liebowitz Social Anxiety Scale (LSAS; Baker, Heinrichs, Kim, and Hoffman, 2002), a mean score of 26 (SD=3.33) on the Fear of Negative Evaluation Questionnaire (FNE; Watson & Friend, 1969), and a mean score of 10.64 (SD=7.18) on the Beck Depression Inventory (BDI; Beck, Rush, Shaw, & Emery, 1979). Patients had a mean age of 28.64 years (SD=3.75) with a mean age of onset of social phobia of 16.07 years (SD=7.64). The mean age at which the memory occurred was 15.18 years (SD=6.99).

### 4.2 Measures

#### 4.2.1 Imagery and memory ratings: distress and vividness

Participants were asked to call their negative image to mind and to dwell on it. They were then asked how distressing the image was on a scale of 0 “not at all” to 100 “extremely.” They were also asked how vivid it was on the same scale. Next they were asked to call to mind their associated memory and to dwell on it. They were asked how distressing and vivid it was on the same scale. Three patients did not complete the image and memory vividness ratings.

#### 4.2.2 Encapsulated belief rating

Participants were asked to rate on a scale of 0 “not at all” to 100 “extremely” how strongly they believed their encapsulated belief, the statement that captured the meaning of their image and memory.

Participants completed two measures of social anxiety.

#### 4.2.3 Social phobia weekly summary scale (SPWSS; Clark et al., 2006)

This six item scale comprises 0–8 ratings of social anxiety, avoidance, self-focused versus externally focused attention, anticipatory processing and postevent rumination.

#### 4.2.4 Social cognitions questionnaire (SCQ; A. Wells, L. Stopa, & D.M. Clark, unpublished)

This 22-item questionnaire asks patients to rate how frequently they have had common social phobia cognitions (e.g., “I will be unable to speak,” “I will sweat/perspire,” “I am foolish”) in the previous week and how much they believed them to be true when they felt anxious. The questionnaire yields two total scores: frequency (of thoughts) and belief (how much they believed the thoughts to be true when they occurred). The total frequency score ranges from 22 to 110 and the total belief score ranges from 0 to 2200. The mean SCQ frequency and belief scores for patients with social phobia at pretreatment in a recent treatment trial (Mortberg, Clark, Sundin, & Wistedt, 2007) were 60 and 950, respectively.

#### 4.2.5 Procedure

Prior to the session, participants completed the SPWSS and the SCQ. The semi-structured interview was then administered. Following this, participants rated how much they believed their encapsulated belief. They also completed imagery and memory ratings. At the end of the memory rescripting, belief ratings and ratings of the vividness and distress of the image and memory were taken again. One week later, participants completed the SPWSS and the SCQ as well as the encapsulated belief, imagery and memory ratings. One of two clinical psychologists, with extensive training in cognitive therapy and imagery rescripting, carried out the procedures with patients.

### 4.3 Results

#### 4.3.1 Within session

Paired samples *t*-tests were performed to compare the difference between the pre- and post-rescripting scores on the following measures: (1) encapsulated belief (2) image distress (3) memory distress (4) image vividness and (5) memory vividness. Effect sizes (*d*) were also calculated to determine the magnitude of effect. Cohen (1988) proposed a threefold classification system of effect sizes: small (.20–.49), medium (.50–.79), and large (.80 and above). Table 1 shows the means and standard deviations of the pre- and post-rescripting scores, the *t* values, significance levels and magnitude of effects. Patients rated the strength of their encapsulated belief as significantly weaker after rescripting compared to before rescripting. They also rated their image and memory as significantly less distressing and less vivid after rescripting compared to before rescripting. All effect sizes were large.

#### 4.3.2 Measures taken 1 week later

Six patients attended a cognitive therapy session before the 1 week follow-up time point and so were excluded from further analyses, leaving eight patients in the follow-up sample. Paired samples *t*-tests were performed to compare the difference between their pre-rescripting scores and 1 week follow-up scores on the social phobia measures (SPWSS and SCQ) and the image and memory measures (encapsulated belief, and image and memory distress and vividness). Table 2 shows the means and standard deviations at the two time points, the *t* values, significance levels and magnitude of effects. On average, patients had significantly lower scores on the SPWSS 1 week after rescripting compared to before rescripting. They also scored significantly lower on the frequency and belief scales of the SCQ 1 week after rescripting compared to before rescripting. Patients appeared to maintain most of their within-session change 1 week later. They rated their encapsulated belief as significantly weaker and their memory as significantly less distressing and less vivid one week after rescripting compared to before rescripting. There was a trend (*p*<.08) for patients to feel less distressed about their recurrent image 1 week after rescripting compared to before rescripting. They also rated their image as less vivid. However, this was not significant (*p*=.111). All effect sizes were medium or large.

## 5 Discussion

This study describes our method of rescripting early memories linked to recurrent negative imagery in patients with social phobia. Further, we looked at the effect of this procedure on the distress, vividness and meaning of patients’ early memories linked to their current negative imagery. We also looked at the effect on social cognitions and other symptoms of social phobia. One session of memory rescripting significantly reduced the distress and vividness of patients’ traumatic social memories as well as their meaning within session and 1 week later. Further, the procedure led to a significant within session reduction in the distress and vividness of patients’ negative images. One week later, there was a trend for patients to rate their images as less distressing, but not less vivid. Finally, on a measure of social cognitions (the SCQ), patients reported having significantly fewer negative social concerns in the previous week (e.g., “I am weird/different;” “people will stare at me”) and reported believing them much less strongly. They also showed significant improvement on the SPWSS, which measures the severity of components of social phobia such as, anxious affect, avoidance, self-focused attention, and anticipatory and postevent processing.

As expected, our sample of patients reported high levels of vividness and distress in relation to their images and memories. This is interesting because vividness has been a cornerstone variable in studies of mental imagery. Yet little is known about its relationship to valence. Experimental studies in non-clinical populations indicate that these variables may be related. For example, Bywaters, Andrade, and Turpin (2004) demonstrated that images of pictures rated as extremely valenced (positively or negatively) were also rated as being more vivid. Several studies in clinical populations suggest high levels of vividness and distress for images associated with current concerns. Pratt, Cooper, and Hackmann (2004) showed that spontaneous and self-generated spider images in people with high spider anxiety were rated as more vivid and distressing than those of participants with low spider anxiety, whilst butterfly images in the two groups did not differ. Studies of body dysmorphic disorder (Osman, Cooper, & Hackmann, 2004) and bulimia nervosa (Somerville, Cooper, & Hackmann, this issue) indicated that whilst images of the self in these disorders were not more frequent than in control groups they were more vivid and distressing. Finally, Hunt et al. (2006) report that blind observer ratings of horror and distress associated with the content of phobic imagery were strongly correlated with participants’ own ratings of the vividness of these same images.

In this study, vividness and distress of both images and memories fell following the intervention. In two studies of intrusive images of memories in PTSD, ratings of vividness, distress, frequency and sense of “nowness” fell steadily in synchrony over several weeks. This followed the start of reliving, restructuring and rescripting of the traumatic memories during treatment. There had been no changes in these variables during the period of assessment (Hackmann et al., 2004; Speckens, Hackmann, Ehlers, & Clark, 2006). The stepped improvement as the weeks went by in these two studies suggests that there may have been further and possibly more stable improvements in the present study on social phobia, had there been more sessions devoted to work on the disturbing memories.

This preliminary, uncontrolled study, like the results of a subsequent controlled study reported elsewhere (Wild et al., in press), supports the view that as well as attending to current symptoms of social phobia it may be worthwhile to look at the possible origins of the problem, and to target key memories that carry toxic meanings for the patient, as they appear to provide input to recurrent images of the self in this disorder. This could be important as the images themselves have been shown to trigger anxiety and safety behaviours, and may thus play a role in maintenance (e.g., Hirsch et al., 2004).

The efficacy of imagery rescripting in this study is consistent with a growing body of research that has used the procedure in cognitive-behavioural treatments for different disorders, including bulimia nervosa (i.e., Cooper et al., 2007; Ohanian, 2001), borderline personality disorder (i.e., Giesen-Bloo et al., 2006), and PTSD (e.g., Smucker & Neiderdee, 1995). Rescripting the identified social phobia related traumatic memories in this study led to dramatic reductions in patients’ beliefs associated with the memories and images, as well as in social phobia cognitions and other symptoms.

There are several aspects of our intervention that may have been helpful. The procedure described here incorporates components of reliving, verbal restructuring and rescripting of the event in imagery. These elements are also components of the protocol for a recently reported effective treatment for PTSD (Ehlers et al., 2005). Imaginal rescripting has also been utilised in isolation, in cases of PTSD where patients had not responded to prolonged exposure (Grunert et al., 2003). Of particular interest are the results of studies like that carried out by Arntz et al. (this issue), which compared imaginal exposure with and without rescripting of memories, and furnishes us with a glimpse of ways in which the effectiveness of various components of the treatment may be examined.

In our study the procedure included asking the patient to vividly evoke the memory three times in total, describing it from various perspectives in the present tense. This repetition is similar to reliving in various CBT programs for PTSD, in which patients are asked to repeatedly go through the traumatic memory. It has been suggested that reliving is effective in PTSD because it allows for reflection and spontaneous cognitive change, as well as habituation (Hackmann, 2005). Foa and Rothbaum (1998) have also observed that reliving allows the patient to come to the possible conclusion that the original negative evaluations were not consistent with all the available evidence. Thus, the reliving aspect of our intervention may have helped patients to come to new conclusions about the earlier event and its current implications.

The verbal restructuring component deliberately prompted patients to consider evidence for and against the meaning of their early memory and image. This helped them to reevaluate how they perceived the event at the time (e.g., were they generally perceived as inferior or unlikable or did it just feel that way?), and how accurate the meaning was in the present (e.g., how do other people actually view them now?). Some patients realised that they were never really worthy of rejection, it just felt like that. Others appreciated that they were rejected in the past, but that this was no longer the case: the meaning of the memory was out of date. In addition, verbal restructuring helped patients to take a less personal view of the event, to see it as meaning (for example) something about the bullies, rather than about themselves.

Finally the patients engaged in imagery rescripting of the early memory. This typically involved imagining entering the scene as an empowered adult who could take effective action, and then subsequently experiencing compassion and nurturance as the younger person. Evoking this vivid imagery from new perspectives is likely to have been another important ingredient in the intervention. Patients may have seen more clearly and felt more strongly that their younger selves were acceptable and deserving of comfort rather than criticism, and that the event had implications about others (e.g., the bullies or critical adults) rather than themselves. Further, some may also have interpreted other people's intentions as more benign than they had previously thought. Hackmann (2005) suggests that experiencing empowerment and compassion in imagery helps patients to reevaluate themselves and the behaviour of others, which can soften the sense of threat.

Overall the process of reviewing and rescripting the traumatic memory reduced the severity of social phobia as reflected in the mean item rating of the SPWSS and also the total belief and frequency scores on the SCQ. It is possible that the procedure led patients to update the meaning of the earlier event and to no longer see it as something toxic with future implications. This may have led them to reevaluate other social concerns which may account for why they had fewer in the week following rescripting and why they believed them less strongly. The SPWSS measures patients’ perceived severity of social anxiety in the previous week as well as their avoidance, self-focused attention, and anticipatory and postevent processing. Regarding change on this measure, it is possible that having learned to see the event differently and to master the event in imagery (in that they intervened, and then soothed the younger self) may have encouraged patients to engage in more social situations, and to do so differently: attending to them more fully once in them, as well as worrying about them less before and afterwards. This study did not look at the direct impact of rescripting on specific processes (e.g., self-focused attention, avoidance, pre- and postevent processing), or the effects on mood, and future research could do this to determine how rescripting mediates change in social phobia symptoms.

This study is a small, exploratory study with several limitations. Firstly, there was no control session. Thus, it is unclear whether imagery rescripting led to change or whether discussion of the early memory without rescripting would have led to change by itself. However, in their recent study, Wild et al. (in press), found that compared to a control session (involving discussion of the memories), imagery rescripting led to significantly greater change in the distress and vividness of patients’ images and memories as well as symptoms of social phobia, both within session and one week later. These results were similar to those in a study of rescripting in bulimia nervosa (Cooper et al., 2007).

Secondly, the sample size is small, particularly after 1 week, in this naturalistic, exploratory study. However, despite this, rescripting did lead to significant within session change, and much significant change when tested after 1 week, which is encouraging, and may stimulate others to do more ambitious studies. Thirdly the intervention was brief, and it might be interesting to try a longer intervention and examine the stability of the results over a longer period of time, as in the study of depressed patients with intrusive memories (Wheatley et al., this issue).

The design of our study does not allow for analysis of the three separate components of our procedure, i.e., reliving, verbal restructuring and imagery rescripting. Thus, it is not possible to conclude which component is the most important or effective. Future studies could compare these components separately and in combination. For example, does the largest shift come from evoking the memory as in the reliving component? Or, is it updating the memory via restructuring and rescripting that shifts beliefs and emotion most effectively? Does verbal restructuring enhance the chance of providing the patient with an alternative and less toxic view of the past? Or, is it sufficient to just prompt for change in the memory image, by asking the patient to view it from another perspective? One could also examine whether interventions that bring about belief change are necessary for symptom reduction, or whether creating other kinds of competing images that contain some of the old elements, but with new features and positive affect might be sufficient to decrease symptomatology (Brewin, 2006).

Finally, one could explore whether identifying and transforming memories that the patient sees as supporting negative beliefs could enhance collaboration and response to other aspects of treatment. Imagery rescripting provides a powerful emotional experience for patients, enhancing compassion for the younger self and reducing the sense of helplessness. It often seems to bring about greater willingness to experiment with dropping safety behaviours and reducing avoidance, as the patient has made the meta-cognitive shift towards seeing the distressing self image as being the product of their own mind, rather than mirroring reality. As one patient most eloquently expressed this shift:

It is as if I have been looking at myself in a mirror for years, and it is only now that I see that it was the mirror that was flawed and not me.

## Figures and Tables

**Table 1:** Means and standard deviations at pre-rescripting and post-rescripting on measures of encapsulated belief, imagery and memory for the total sample

Measure	Total sample (*N*=14)				Analysis			
	Pre-rescripting		Post-rescripting		Paired samples *t*-test			Effect size
	Mean	SD	Mean	SD	*t*	df	*p*	*d*
Encapsulated belief	75.71	23.73	22.14	18.90	7.54	13	.001	2.51
Image distress	54.29	24.08	20.71	22.69	4.97	13	.001	1.44
Memory distress	66.07	22.03	23.57	25.53	4.93	13	.001	1.79
Image vividness	60.91	27.73	32.27	25.82	3.32	10	.008	1.07
Memory vividness	68.64	24.09	45.45	30.78	2.92	10	.015	.86

**Table 2:** Means and standard deviations at pre-rescripting and 1 week post-rescripting on social anxiety, image and memory measures

Measure	Follow-up sample (*n*=8)				Analysis			
	Pre-rescripting		One week post-rescripting		Paired samples *t*-test			Effect size
	Mean	SD	Mean	SD	*t*	df	*p*	*d*
Social phobia weekly summary scale	5.02	1.16	4.02	1.46	2.61	7	.035	0.76
Social cognitions frequency	58.38	11.87	47.50	9.29	3.61	7	.009	1.03
Social cognitions total belief score	815.63	320.04	605.63	275.26	2.83	7	.025	0.71
Encapsulated belief	74.88	27.55	33.38	28.11	3.31	7	.013	1.49
Image distress	55.00	28.28	27.50	26.59	2.02	7	.083	1.00
Memory distress	74.38	21.62	3.75	7.44	8.67	7	.001	4.86
Image vividness	65.00	32.09	35.00	23.45	1.94	5	.111	1.08
Memory vividness	82.50	19.93	30.00	31.62	5.18	5	.004	2.04

## Footnotes

1 Details of cases have been slightly changed to ensure anonymity.

## References

[bib1] Alden L.E., Wallace S.T. (1995). Social phobia and social appraisal in successful and unsuccessful social interactions. Behaviour Research and Therapy.

[bib2] American Psychiatric Association (2000). Diagnostic and statistical manual of mental disorders.

[bib3] Arntz, A., Tiesema, & Kindt, M. (this issue). Treatment of PTSD: A comparison of imaginal exposure with and without imagery rescripting. *Journal of Behavior Therapy and Experimental Psychiatry*. 10.1016/j.jbtep.2007.10.006 18005935

[bib4] Arntz A., Weertman A. (1999). Treatment of childhood memories: Theory and practice. Behaviour Research and Therapy.

[bib5] Baker S.L., Heinrichs N., Kim H.J., Hoffman S.G. (2002). The Liebowitz Social Anxiety Scale as a self-report instrument: A preliminary psychometric analysis. Behaviour Research and Therapy.

[bib6] Beck A.T. (1976). Cognitive therapy and the emotional disorders.

[bib7] Beck A.T., Emery G., Greenberg R. (1985). Anxiety disorders and phobias.

[bib8] Beck A.T., Rush A.J., Shaw B.F., Emery G. (1979). Cognitive therapy of depression.

[bib9] Beck J.S. (1995). Cognitive therapy: Basics and beyond.

[bib10] Brewin C.R. (2006). Understanding cognitive behaviour therapy: A retrieval competition account. Behaviour Research and Therapy.

[bib11] Brown T.A., Di Nardo P.A., Barlow D.H. (1994). Anxiety disorders interview schedule for DSM-IV.

[bib12] Bywaters M., Andrade J., Turpin G. (2004). Determinants of the vividness of visual imagery: The effects of delayed recall, stimulus affect and individual differences. Memory.

[bib13] Clark D.M., Wells A., Heimberg R., Liebowitz M., Hope D.A., Schneier F.R. (1995). A cognitive model of social phobia. Social phobia: Diagnosis, assessment and treatment.

[bib14] Clark D.M., Ehlers A., Hackmann A., McManus F., Fennell M., Grey N. (2006). Cognitive therapy versus exposure and applied relaxation in social phobia: A randomized controlled trial. Journal of Consulting and Clinical Psychology.

[bib15] Cohen J. (1988). Statistical power analysis for the behavioural sciences.

[bib16] Coles M.E., Turk C.L., Heimberg R.G. (2002). The role of memory perspective in social phobia: Immediate and delayed memories for role-played situations. Behavioural and Cognitive Psychotherapy.

[bib17] Coles M.E., Turk C.L., Heimberg R.G., Fresco D.M. (2001). Effects of varying levels of anxiety within social situations: Relationship to memory perspective and attributions in social phobia. Behaviour Research and Therapy.

[bib18] Conway M., Meares K., Standart S. (2004). Images and goals. Memory.

[bib19] Cooper, M., Todd, G., & Turner, H. (2007). The effects of using imagery to modify core emotional beliefs in bulimia nervosa: An experimental pilot study. *Journal of Cognitive Psychotherapy*, *21*, 117–121.

[bib20] Edwards D.J. (1990). Cognitive therapy and the restructuring of early memories through guided imagery. Journal of Cognitive Psychotherapy: An International Quarterly.

[bib21] Ehlers A., Clark D.M. (2000). A cognitive model of posttraumatic stress disorder. Behaviour Research and Therapy.

[bib22] Ehlers A., Clark D.M., Hackmann A., McManus F., Fennell M. (2005). Cognitive therapy for posttraumatic stress disorder: Development and evaluation. Behaviour Research and Therapy.

[bib23] Ehlers A., Hackmann A., Michael T. (2004). Intrusive re-experiencing in posttraumatic stress disorder: Phenomenology, theory and therapy. Memory.

[bib24] First M.B., Gibbon M., Williams J.B., Spitzer R.L. (1995). The structured clinical interview for DSM-IV.

[bib25] Foa E.B., Rothbaum B.O. (1998). Treating the trauma of rape: Cognitive-behavioural therapy for PTSD.

[bib26] Giesen-Bloo J., van Dyck R., Spinhoven P., van Tilburg W., Dirksen C., van Asselt T. (2006). Outpatient psychotherapy for borderline personality disorder: A randomized clinical trial of schema focused therapy versus transference focused psychotherapy. Archives of General Psychiatry.

[bib27] Grunert B.K., Smucker M.R., Weis J.M., Rusch M.D. (2003). When prolonged exposure fails: Adding an imagery-based cognitive restructuring component in the treatment of industrial accident victims suffering from PTSD. Cognitive and Behavioral Practice.

[bib28] Hackmann, A. (1998). Working with images in clinical psychology. In A. Bellack & M. Hersen (Eds.), Comprehensive Clinical Psychology: Vol. 6. (pp. 301–318).

[bib29] Hackmann A., Gilbert P. (2005). Compassionate imagery in the treatment of early memories in Axis I anxiety disorders. Compassion: Conceptualisations, research and use in psychotherapy.

[bib30] Hackmann A., Clark D.M., McManus F. (2000). Recurrent images and early memories in social phobia. Behaviour Research and Therapy.

[bib31] Hackmann A., Ehlers A., Speckens A., Clark D.M. (2004). Characteristics and content of intrusive memories in PTSD and their changes with treatment. Journal of Traumatic Stress.

[bib32] Hackmann A., Surawy C., Clark D.M. (1998). Seeing yourself through others’ eyes: A study of spontaneously occurring images in social phobia. Behavioural and Cognitive Psychotherapy.

[bib33] Hirsch C.R., Clark D.M., Mathews A., Williams R. (2003). Self-images play a causal role in social phobia. Behaviour Research and Therapy.

[bib34] Hirsch C.R., Mathews A., Clark D.M., Williams R., Morrison J.A. (2006). The causal role of negative imagery in social anxiety: A test in confident public speakers. Journal of Behavior Therapy an Experimental Psychiatry.

[bib35] Hirsch C.R., Meynen T., Clark D.M. (2004). Negative self-imagery in social anxiety contaminates social interactions. Memory.

[bib36] Hunt M., Bylsma L., Brock J., Fenton M., Goldberg A., Miller R. (2006). The role of imagery in the maintenance and treatment of snake fear. Journal of Behavior Therapy and Experimental Psychiatry.

[bib37] Layden M.A., Newman C.F., Freeman A., Morse S.B. (1993). Cognitive therapy of borderline personality disorder.

[bib38] Levis D.J., Goldstein A., Foa E.B. (1980). Implementing the technique of implosive therapy. Handbook of behavioral interventions.

[bib39] Mortberg E., Clark D.M., Sundin O., Wistedt A. (2007). Intensive group cognitive treatment and individual cognitive therapy vs. treatment as usual in social phobia: A randomized controlled trial. Acta Psychiatrica Scandinavia.

[bib40] Mulkens S., de Jong P.J., Dobbelaar A., Bogels S. (1999). Fear of blushing: Fearful preoccupation irrespective of facial coloration. Behaviour Research and Therapy.

[bib41] Ohanian V. (2001). Imagery rescripting within cognitive behavior therapy for bulimia nervosa: An illustrative case report. International Journal of Eating Disorders.

[bib42] Osman S., Cooper M., Hackmann A. (2004). Spontaneously occurring images and early memories in people with body dysmorphic disorder. Memory.

[bib43] Pratt D., Cooper M.J., Hackmann A. (2004). Imagery and its characteristics in people who are anxious about spiders. Behavioural and Cognitive Psychotherapy.

[bib44] Rachman S.J. (1980). Emotional processing. Behaviour Research and Therapy.

[bib45] Rapee R., Lim L. (1992). Discrepancy between self and observer ratings of performance in social phobics. Journal of Abnormal Psychology.

[bib46] Rapee R.M., Abbott M.J. (2006). Mental representation of observable attributes in people with social phobia. Journal of Behavior Therapy and Experimental Psychiatry.

[bib47] Rapee R.M., Heimberg R. (1997). A cognitive-behavioral model of anxiety in social phobia. Behaviour Research and Therapy.

[bib48] Smucker M.R., Neiderdee J. (1995). Treating incest-related PTSD and pathogenic schemas through imaginal exposure and rescripting. Cognitive and Behavioral Practice.

[bib49] Somerville, K., Cooper, M., & Hackmann, A. (this issue). Spontaneous imagery in women with bulimia nervosa: An investigation into content, characteristics and links to childhood memories. *Journal of Behavior Therapy and Experimental Psychiatry*. 10.1016/j.jbtep.2007.09.003 17988650

[bib50] Speckens A., Hackmann A., Ehlers A., Clark D.M. (2006). Changes in intrusive memories associated with imaginal reliving in post-traumatic stress disorder. Journal of Anxiety Disorders.

[bib51] Stopa L., Clark D.M. (1993). Cognitive processes in social phobia. Behaviour Research and Therapy.

[bib52] Vassilopoulos S. (2005). Social anxiety and the effects of engaging in mental imagery. Cognitive Therapy and Research.

[bib53] Watson D., Friend R. (1969). Measurement of social-evaluative anxiety. Journal of Consulting and Clinical Psychology.

[bib54] Weertman, A., & Arntz, A. (in press). Effectiveness of treatment of childhood memories in cognitive therapy for personality disorders: A controlled study contrasting methods focusing on the present and methods focusing on childhood memories. *Behaviour Research and Therapy*. 10.1016/j.brat.2007.02.013 17462588

[bib55] Weitzman B. (1967). Behaviour therapy and psychotherapy. Psychological Review.

[bib56] Wells A., Clark D.M., Ahmad S. (1998). How do I look with my minds eye? Perspective taking in social phobic imagery. Behaviour Research and Therapy.

[bib57] Wheatley, J., Brewin, C. R., Patel, T., & Hackmann, A. (this issue). “I’ll believe it when I can see it”: Imagery re-scripting of intrusive sensory memories in depression. *Journal of Behavior Therapy and Experimental Psychiatry*. doi:10.1016/j.jbtep.2007.08.005. 10.1016/j.jbtep.2007.08.005 17915192

[bib58] Wild, J., Hackmann, A., & Clark, D. M. (in press). Rescripting early memories linked to negative images in social phobia: A pilot study. *Behavior Therapy.* 10.1016/j.beth.2007.04.003 PMC2270350 18328869

[bib59] Young J.E., Klosko J.S., Weishhaar M.E. (2003). Schema therapy: A practitioner's guide.

